# Legume Green Manure Further Improves the Effects of Fertilization on the Long-Term Yield and Water and Nitrogen Utilization of Winter Wheat in Rainfed Agriculture

**DOI:** 10.3390/plants14162476

**Published:** 2025-08-09

**Authors:** Xiushuang Li, Juan Chen, Jianglan Shi, Xiaohong Tian

**Affiliations:** 1 Key Laboratory of Land Resources Evaluation and Monitoring in Southwest, Ministry of Education/College of Geography and Resource Science, Sichuan Normal University, Chengdu 610068, China; lixiushuang@sicnu.edu.cn; 2 College of Natural Resources and Environment, Northwest A&F University/Key Lab of Plant Nutrition and the Agri-Environment in Northwest China, Ministry of Agriculture and Rural Affairs, Yangling 712100, China; shijl81@nwsuaf.edu.cn; 3 Center for Technical Support of Nuclear Emergency in Sichuan, Institute of Radiation Test and Protection of Nuclear Industry in Sichuan, Chengdu 610068, China; chenjuan2015@foxmail.com

**Keywords:** legume green manure, winter wheat yield, water use efficiency, plastic film mulching, rainfed agriculture

## Abstract

**Context:** To revive the practice of planting legume green manure (GM) in the fallow period in rainfed agricultural areas, it is essential to demonstrate the benefits of this practice on the yields and water use efficiency (WUE) of subsequent crops, especially when integrating with optimized water and fertilizer management. **Objectives:** We conducted a field experiment to determine the positive effects of planting legume GM in the summer fallow on the yield, WUE, and nitrogen uptake efficiency (NupE) of subsequent winter wheat, which was grown with plastic film mulching and integrated fertilization in the Loess Plateau of China. **Methods:** A split-plot-designed experiment was arranged with two main treatments, namely (1) wheat planting followed by GM planting in the summer fallow (GM) and (2) conventional wheat monoculture followed by bare land summer fallow (BL), and three sub-treatments: (1) control treatment without any chemical fertilizer (Ct), (2) application of chemical N, P, and K as basal fertilizer (B), and (3) application of basal fertilizer plus wheat straw return (BS). **Results:** In the initial two years, even in a dry year, GM did not decrease the soil water content and storage (0–200 cm layer) during the subsequent winter wheat season, relative to BL. But in the third and fourth years, GM increased the grain yield of winter wheat by 3.2% and 3.8%, respectively. B and BS increased the grain yield of winter wheat by 14.4% and 22.2%, respectively, during the third experimental year, and by 12.7% and 19.4% during the fourth experimental year, primarily through increasing the population density of winter wheat. The increase in the grain yield contributed to a higher WUE of winter wheat. In the third year, GM increased the water consumption (WC) and WUE of wheat by 2.4% and 1.7%, respectively, though they were far lower than B (8.3% and 5.6%) and BS (10.4% and 10.7%). B and BS resulted in a higher yield and N nutrition than GM alone, but GM combined with B and BS resulted in the highest yield and N nutrition, thus greatly decreasing the NupE and increasing N productivity. **Conclusions:** Planting legume GM in the fallow can further increase the long-term yield, WUE, and N utilization of winter wheat when integrated with chemical fertilization and wheat straw return in rainfed agriculture. **Implications:** Our study yields new insights into the agronomic benefits of legume GM application in semi-arid or analogous rainfed agroecosystems and underscores the critical role of water conservation in ensuring dryland agricultural production, particularly in regions undergoing optimization of fertilization.

## 1. Instruction

Rainfed agriculture is common in dryland agroecosystems, accounting for approximately three quarters of cropland worldwide [1,2]. In typical rainfed agriculture, crop yields depend on natural rainfall as the sole water resource and thus are greatly restricted by unfavorable weather conditions [3,4]. Therefore, agronomic practices that improve the water use efficiency (WUE) of crops have been increasingly adopted to achieve higher yields [5,6]. Agronomic practices that improve WUE include ground mulching, plastic film mulching, conservation tillage, and partial substitution of chemical fertilizers with organic manure. These practices help conserve soil moisture by regulating the physical and chemical properties of the soil. Plastic film mulching has particularly been favored because of its great efficacy for improving both grain yield and WUE in rainfed agriculture areas [6,7,8,9].

Winter wheat (*Triticum aestivum* L.) is a major food crop worldwide and is mainly planted in dryland agroecosystems [2,10]. Winter wheat occupies almost half of the cropland in the Loess Plateau of China, where rainfed agriculture without any irrigation is typical [11]. The annual precipitation in the Loess Plateau ranges from 250 to 650 mm, with only 40% falling during the winter wheat season, from October to June of the following year [6,7]. Moreover, there is great inter-annual variation and an uneven distribution of rainfall during the wheat growing season, resulting in frequent droughts as well as unstable and low wheat yields [9,12]. Improving soil water conservation and WUE is important for achieving high yields of dryland winter wheat. Winter wheat is conventionally monocultivated and followed by about three months of summer fallow each year, during which time approximately 60% of annual precipitation will occur and the bare land can help store water in the soil for the subsequent winter wheat growth [8,13,14]. In addition, plastic film mulching is widely practiced throughout the whole winter wheat growing season in this region, as it can significantly reduce evaporation, improve soil moisture, and increase crop yields [6,9,15].

In addition to water limitations, soil quality degradation is also an obstacle faced by dryland agriculture. Soil erosion in drylands is typically caused by wind and/or water [14]. Moreover, with the rapid development of the chemical fertilizer industry, excessive applications of chemical fertilizer, especially nitrogen (N) fertilizer, as well as insufficient applications of organic matter, exacerbate soil quality degradation, resulting in a fragile physical structure and low organic matter content, as well as a low N utilization efficiency [13,16]. Soil quality degradation occurs extensively in dryland areas worldwide, including in the Loess Plateau of China. Crop production in drylands is also limited by a low nutrient supply [14,15]. The lack of organic input can induce soil organic matter depletion and cause serious environmental problems [15]. Optimizing fertilizer management and increasing the organic fertilizer input is urgent for dryland agriculture in the Loess Plateau to improve soil quality, maintain sustainable productivity, and increase nutrient utilization efficiency [17].

The return of crop straw to soil as an organic amendment is commonly used to improve soil fertility [15,17,18]. To avoid the potential N immobilization caused by large inputs of organic carbon, crop straw application has been effectively optimized by integrating it with chemical fertilizers [19,20]. Researchers have also noted that straw return can increase soil organic carbon content and microbial activity and alleviate the soil degradation caused by intensive and continuous tillage [18,19]. However, crop straw return alone does not necessarily improve soil fertility and quality over the short term, particularly in fields where there has been insufficient organic fertilizer input for decades [17,19].

Recent research has shown that the conventional summer fallow period, which was typically used to store water in the soil, actually does not effectively store water and can lead to further degradation of fertilized soil due to water and/or wind erosion and cause low utilization of natural light and heat resources, thus posing a threat to sustainable production [10,13,14]. Therefore, in addition to increasing organic input, it is critical to develop agronomic practices that adequately use the natural resources during the rainy summer.

Planting a legume green manure (GM) crop is an alternative agronomic option that could help crops exploit the natural resources of light and heat during the fallow period. Thus, this could play a vital role in ecological restoration and increasing soil fertility in degraded agroenvironments [21,22,23]. Legume GM is primarily used as a soil organic amendment and has been traditionally used in agriculture in China for a long time [23,24]. Recently, legume GM has received considerable attention for its contribution to the dual goals of food security and sustainable agriculture; it can produce substantial biomass and lead to nutrient accumulation, especially via N fixation [16,23,25,26]. Incorporation of legume GM into the field can maintain soil fertility and biological activity, supplement mineral N, and improve the grain yield of subsequent cereal crops [27,28,29]. Consequently, planting legume GM during the summer fallow and incorporating it into the soil at a suitable time could maximize benefits from natural resources and establish sustainable agriculture production in the Loess Plateau.

However, cover crops, including legume GM, have not been planted during the fallow period in many semi-arid and arid regions of China because it was thought that they might exacerbate soil water depletion and negatively affect the water supply for subsequent crops [30,31]. Studies have had varying results; for example, wheat yield decreased substantially when the preceding fallow period was replaced with legume crops in the Central Great Plains of the USA [32]. In the Loess Plateau, studies showed that planting a forage crop or legume GM during the fallow period did not greatly influence the soil water storage to be used by subsequent winter wheat, but it could actually increase both the WUE and yield of winter wheat when no water conservation method was practiced [31]. The effects of legume GM on the subsequent crops among various cropping systems are still unclear. In addition, current studies on the application of legume GM have mostly focused on its effect on the fertilizer reduction or N fertilizer efficiency of subsequent crops [20,22,23,33]. The effects of legume GM combined with other fertilizer strategies on the WUE as well as N uptake efficiency of subsequent crops have rarely been reported, especially on the basis of a unified water conservation practice. To widely revive planting legume GM in the rainy summer fallow in the Loess Plateau, it is essential to investigate the effect of planting legume GM on the productivity and WUE of subsequent winter wheat under different water conservation strategies, as well as the nutrient utilization efficiency under organic fertilizer management.

Based on the above background, we hypothesized that GM would not greatly affect soil water utilization during the subsequent winter wheat season under a water conservation strategy and would somewhat improve the yield, WUE, and N utilization under optimal fertilizer management. We conducted a 4-year field experiment in which winter wheat was grown using plastic film mulching in the Loess Plateau and compared planting a legume GM crop during the fallow period with wheat monocropping under three fertilizer/organic amendment treatments. The soil water supply of winter wheat was evaluated utilizing dynamic observations obtained from various wheat growing stages. We also explored the impacts of planting GM on the yield and N uptake efficiency of subsequent winter wheat when integrating with increased straw return. Our results provide a theoretical basis to guide field management strategies on the planting of GM in dryland agroecosystems.

## 2. Results

### 2.1. Wheat Yield and Yield Components

**Figure 1:**
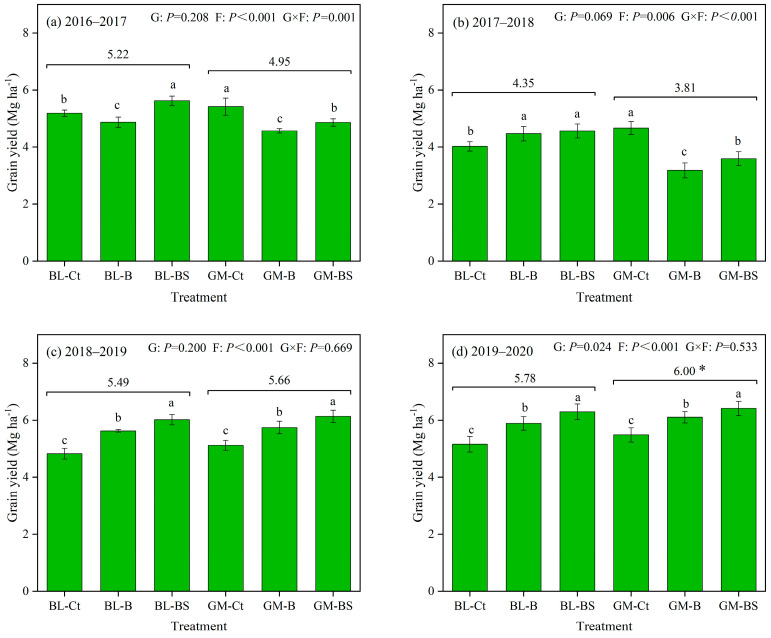
Grain yield of the winter wheat as affected by planting regime (G) and fertilization treatment (F) during the four wheat seasons: 2016–2017 (**a**), 2017–2018 (**b**), 2018–2019 (**c**) and 2019–2020 (**d**). Ct, B, and BS represent the control (no fertilization), basal fertilization with NPK fertilizers, and basal fertilization plus wheat straw return, respectively. GM represents planting GM and incorporation during summer fallow before the wheat season, compared with conventional wheat cultivation after a bare land fallow (BL). Two-way (G and F) ANOVA and a LSD test were used for each year to determine the significances among treatments. Filled boxes indicate the mean values with their standard errors as error bars (mean ± SE; n = 3). * indicates a significant difference (*p* < 0.05) between the two main treatments affected by G. Different lower-case letters indicate significant differences (*p* < 0.05) between the three fertilization patterns.

The winter wheat yield greatly differed across the four experimental years (Figure 1). In the initial two years, the wheat yield was not significantly affected by G (Figure 1a,b, *p* < 0.05). Relative to BL, GM decreased the wheat yield by 5.6% and 14.1% on average during the first and second experimental years, respectively. There were also no clear effects of fertilization on wheat yield in these two years (Figure 1a,b). In contrast, GM increased the wheat yield in both the third and fourth years, and the increase in the fourth year was significant (Figure 1c,d, *p* < 0.05). Compared with BL, GM increased the wheat yield by 3.2% on average in the third year, and significantly increased the wheat yield (by 3.8%) in the fourth year. Meanwhile, compared with the controls (BL-Ct and GM-Ct), basal fertilization (BL-B and GM-B) significantly increased the wheat yield by an average of 14.4% and 12.7% in the third and fourth years, respectively. Basal fertilization plus straw return (BL-BS and GM-BS) significantly increased the wheat grain yield by an average of 22.2% and 19.4% in the last two years, respectively, relative to the controls (Figure 1c,d, *p* < 0.05).

Consistent with the grain yield, the positive effect of planting GM and fertilization on the aboveground biomass of winter wheat emerged beginning in the third test year (2018–2019) (Table 1). Compared with BL, GM increased the wheat biomass by an average of 9.8% during the third season. In contrast, when compared with the controls, BL-B and GM-B significantly increased the wheat biomass by an average of 11.9% in the third season (*p* < 0.05). Furthermore, BL-BS and GM-BS significantly increased the wheat biomass by an average of 21.4% (*p* < 0.05). Also, BL-BS and GM-BS yielded the highest number of spikes per hectare among all the six treatments (*p* < 0.05), followed by the BL-B and GM-B treatments, during the third year. In addition, relative to the controls, BL-BS and GM-BS increased the 1000-grain weight (*p* < 0.05), which did not significantly differ from BL-B and GM-B during the third year. On the contrary, with the exception of a slight decrease in spike number during 2017–2018, GM did not significantly affect the yield components of wheat across the first three experimental years, relative to BL. The number of kernels per spike and the harvest index varied substantially across all experimental years, and were not significantly affected by the different treatments even during the third experimental year.

### 2.2. Soil Water Storage and WUE During Wheat Seasons

**Figure 2:**
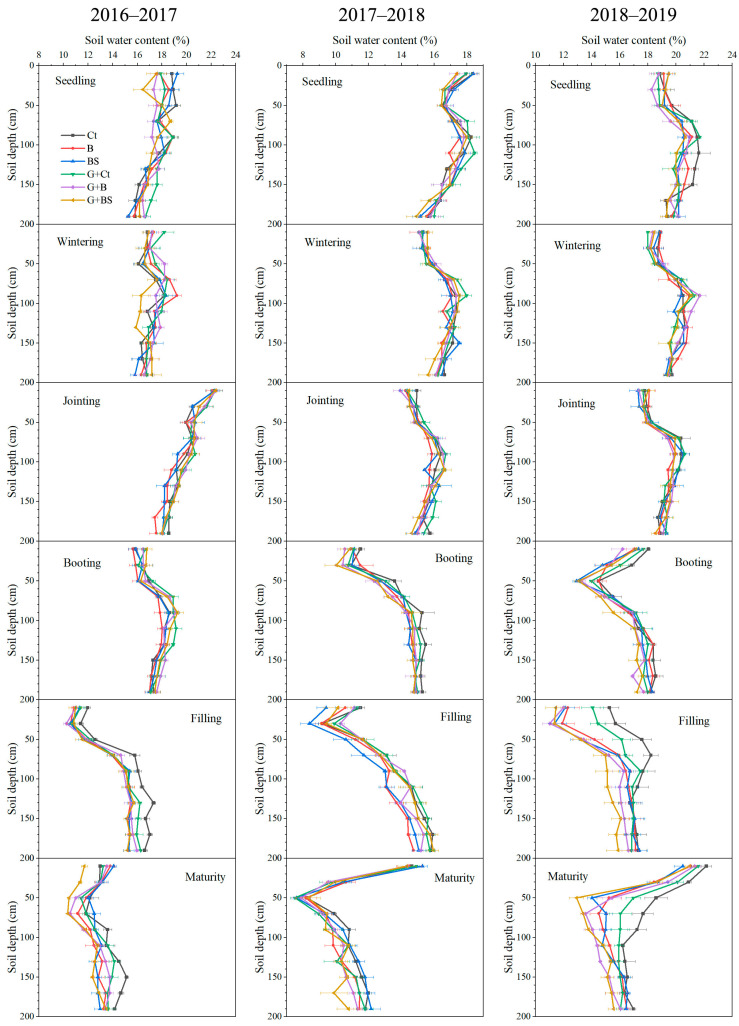
Soil water content in the upper 0–200 cm layer at different growing stages of winter wheat among treatments across 2016–2017, 2017–2018, and 2018–2019. Ct, B, and BS represent the control (no fertilization), basal fertilization with NPK fertilizers, and basal fertilization plus wheat straw return, respectively. GM represents planting GM and incorporation during summer fallow before the wheat season, compared with conventional wheat cultivation after a bare land fallow (BL). Data are shown as the means with the standard errors (mean ± SE; n = 3).

The soil water content (SWC) in the upper 0–200 cm layer at different wheat growing stages (seedling, wintering, jointing, booting, filling, and maturity) during the first three experimental seasons is shown in Figure 2. In line with the winter wheat yield, SWC exhibited differences among various years and among various treatments. In 2016–2017, the SWC did not obviously differ among treatments in the early stages of winter wheat growing, and only the BL-Ct treatment had a slightly higher SWC in the deep soil layer in the late stages (filling and maturity) relative to the other treatments. During 2017–2018, the SWC in the 0–200 cm soil layer did not exhibit obvious differences among the treatments across all stages of winter wheat growing. However, the SWC of the 0–120 cm layer exhibited obvious differences among treatments in the late filling and maturity stages of winter wheat during 2018–2019. In these stages, the BL-Ct treatment had the highest SWC in the 0–120 cm soil layer, followed by the GM-Ct treatment, relative to the other treatments.

**Figure 3:**
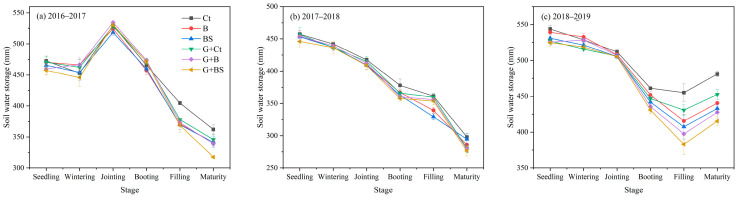
Soil water storage in the 0–200 cm soil layer during the winter wheat growing seasons of 2016–2017 (**a**), 2017–2018 (**b**), and 2018–2019 (**c**). Ct, B, and BS represent the control (no fertilization), basal fertilization with NPK fertilizers, and basal fertilization plus wheat straw return, respectively. GM represents planting GM and incorporation during summer fallow before the wheat season, compared with conventional wheat cultivation after a bare land fallow (BL). Data are shown as the means with the standard errors (mean ± SE; n = 3).

The soil water storage (SWS) in the 0–200 cm layer in different wheat growing seasons (2016–2017, 2017–2018, and 2018–2019) at different wheat growing stages is shown in Figure 3. Consistent with SWC, differences in SWS in the 0–200 cm soil layer primarily occurred at the late stages (filling and maturity) of wheat growing during 2018–2019 (Figure 3c). Specifically, when compared with BL, GM resulted in relatively lower SWS mainly during the late growing period of winter wheat.

**Figure 4:**
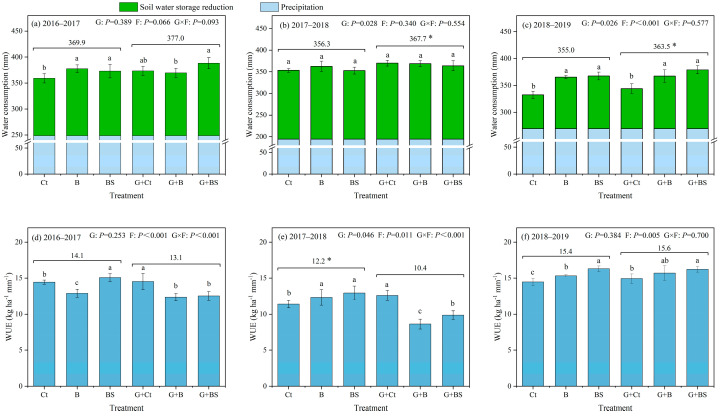
The water consumption (**a**–**c**) and water use efficiency (WUE) (**d**–**f**) as affected by planting regimes (G) and fertilization patterns (F) during the winter wheat growing seasons of 2016–2017, 2017–2018, and 2018–2019. Ct, B, and BS represent the control (no fertilization), basal fertilization with chemical NPK fertilizers, and basal fertilization plus wheat straw return. GM represents planting GM and incorporation during summer fallow before the wheat season (BL). Two-way (G and F) ANOVA and an LSD test were used for each year to determine the significances among treatments. Filled boxes indicate the mean values with the standard errors as error bars (mean ± SE; n = 3). * indicates a significant difference (*p* < 0.05) between the two main treatments affected by G. Different lower-case letters indicate significant differences (*p* < 0.05) between the three fertilization patterns.

**Figure 5:**
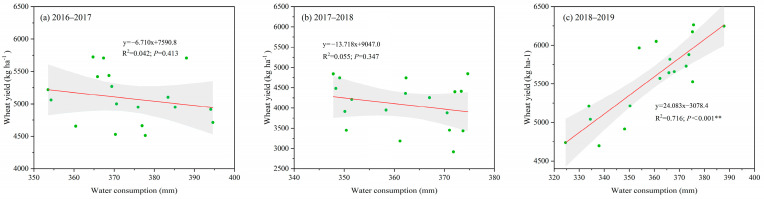
Relationship between cumulative water consumption (mm) and grain yield (kg ha^−1^) during winter wheat growing seasons of 2016–2017 (**a**), 2017–2018 (**b**), and 2018–2019 (**c**). Pearson correlation analysis was used for each year to determine relationships. The gray areas are the confidence interval (at 95% level) of the linear functions. ** indicates a highly significant relationship (*p* < 0.01).

The cumulative water consumption (WC) and water use efficiency (WUE) of winter wheat during 2016–2017, 2017–2018, and 2018–2019 are shown in Figure 4. Neither G nor F had a large effect on WC during 2016–2017. With the exception of a slight increase caused by G (*p* < 0.05), the fertilization patterns also did not affect WC during 2017–2018. However, both G and F significantly altered WC during 2018–2019 (*p* < 0.05). Specifically, relative to BL, GM promoted soil water storage reduction and a reduction in the total WC of wheat seasons by 9.9% and 2.4% on average during 2018–2019, respectively (Figure 4b,c). In addition, the treatments with chemical fertilization (BL-B, BL-BS, GM-B, and GM-BS) increased WC during 2018–2019 relative to the controls without chemical fertilization (Figure 4c). In contrast, the WUE differed substantially among treatments due to it being largely dependent on the grain yield (Figure 4d–f). Although GM induced a slight decrease in WUE during 2017–2018 (*p* < 0.05), it conversely increased the WUE by 1.7% during 2018–2019 relative to BL. During 2018–2019, the treatments with basal fertilization plus wheat straw return (BL-BS and GM-BS) resulted in the highest WUE (16.3 and 16.2 kg ha^−1^mm^−1^), followed by basal fertilization treatments (BL-B and GM-B, 15.3 and 15.7 kg ha^−1^mm^−1^) and controls (14.5 and 14.9 kg ha^−1^mm^−1^) (*p* < 0.05). WUE generally increased with increasing grain yield (Figure 1 and Figure 4). In addition, except for the early two years, a highly significant (*p* < 0.01) correlation was also observed between the WC and wheat yield during 2018–2019, showing that a higher WC was consistent with a higher wheat yield (Figure 5).

### 2.3. N Concentrations and N Use Efficiency of Winter Wheat

The N concentrations in both the grain and straw of the winter wheat of the different treatments across the four experimental years are shown in Table 2. Relative to BL, GM generally increased the N concentrations in both the grain and straw of winter wheat across the four experimental years; these increases ranged from 2.2% to 6.4% and from 2.3% to 11.9% in the grain and straw, respectively. These differences were significant (*p* < 0.05) during the last two experimental years (2018–2019 and 2019–2020). Specifically, GM increased the N concentration in wheat grain by 6.4% and 5.8%, respectively, during these two years, and increased the N concentration in wheat straw by 10.4% and 8.0%, respectively. In contrast, F significantly affected the N concentrations in both the grain and straw of wheat across the four experimental years. In general, the N concentrations in both grain and straw followed the order BS > B > Ct, regardless of G. Specifically, during 2018–2019 and 2019–2020, B treatments (BL-B and GM-B) increased the N concentration in wheat grain by 4.1% and 3.9% on average, respectively, and increased the N concentration in wheat straw by 17.5% and 17.4%, respectively, relative to the Ct treatments. Additionally, BS treatments (BL-BS and GM-BS) increased the N concentration in wheat grain by 9.1% and 9.2% on average, respectively, and increased the N concentration in wheat straw by 24.0% and 22.1%, respectively. GM-BS resulted in the highest N concentrations in both the grain and straw across the four experimental years.

We found clear differences in the cumulative N inputs just before winter wheat seeding among the treatments across the four experimental years (Table 3). The cumulative N input, which included the application of chemical N fertilizer, the GM planting during the preceding summer fallow, the rated wheat straw return, and the remaining wheat straw from the last wheat season, atmospheric deposition, and wheat seeds, ranged from 25.1 to 329 kg ha^−1^ throughout the four experimental years. Specifically, the added N input that derived from planting legume GM in the fallow period was 74.3–88.8 kg ha^−1^ across the four experimental years, averaging 81.9 kg ha^−1^ yr^−1^.

The NupE of winter wheat was significantly decreased by G across the four experimental years (*p* < 0.05); on average, the decrease ranged from 132% to 158%, when compared with the conventional cultivation of winter wheat (Table 4). The NUE under GM also decreased across the four experimental years in comparison with BL, and this effect was significant (*p* < 0.05) during the last two years (2018–2019 and 2019–2020). In these two years, the NUE under GM decreased by 10.0% and 6.8%, respectively, relative to BL. F significantly affected both the NupE and NUE across the four experimental years (*p* < 0.05). The NupE and NUE followed the order BS < B < Ct, regardless of G. Moreover, the BL-Ct, BL-B, and GM-Ct treatments almost all resulted in a NupE of winter wheat higher than 1 across the four experimental years. GM-BS generally resulted in both the lowest NupE and NUE among the six treatments across the four experimental years.

## 3. Discussion

### 3.1. Changes in Winter Wheat Yield

**Figure 6:**
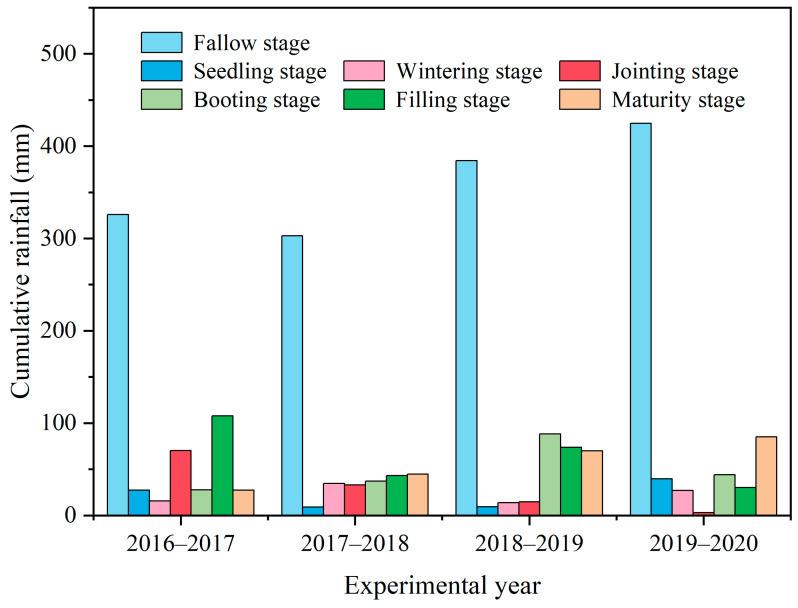
The distribution of annual precipitation during the fallow stage and various wheat growing stages, respectively, during the four experimental years.

**Figure 7:**
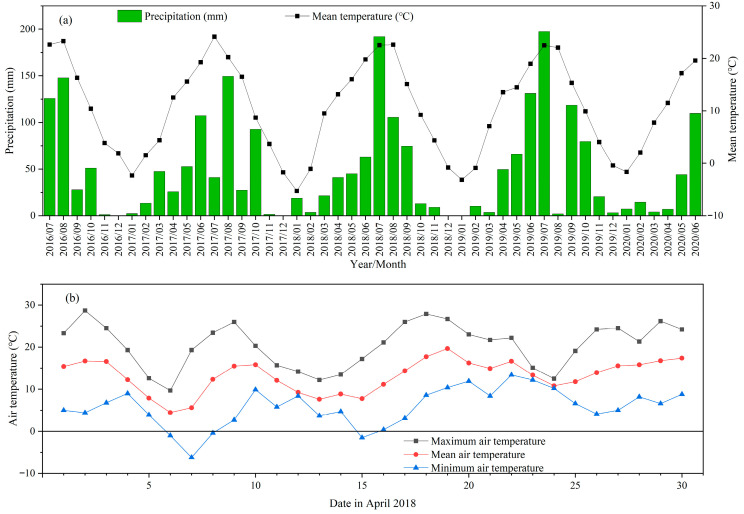
The precipitation (mm) and temperature (°C) during the experimental period in the experimental area: (**a**) shows the monthly cumulative precipitation and mean temperature from July 2016 to June 2020; (**b**) shows the daily air temperature in April 2018 in which extreme weather (frost) occurred.

Organic amendment addition (including legume GM) and fertilization can improve soil fertility and quality, increasing crop growth. However, in this study, neither legume GM nor fertilization increased the grain yield of winter wheat during the initial two years, and GM even slightly decreased the yield in some treatments (Figure 1). This might be largely attributed to the spatial heterogeneity of the original soil fertility and extreme weather conditions [15]. In specific, the second year (2017–2018) was classified as a dry year for winter wheat in this region, as rainfalls occurring at summer fallow and the seedling stages were both relatively low, which could be unfavorable for the available tillering (Figure 6). This can also be evidenced by the lowest spike density relative to other years (Table 1). Moreover, a frost occurred in early April 2018 and lasted 3 days just before the winter wheat booting stage, inducing a relatively low kernel amount (Figure 7 and Table 1). It is possible that GM and fertilization initially promoted the growth of wheat, but then these high-growth plants were more susceptible to damage from the poor weather conditions [15], leading to overall lower yields in the treatments with both GM and fertilization in 2017–2018 (Figure 1). Although the planting of GM in the fallow period can increase soil water consumption and be unfavorable for subsequent crop growth, we supposed this effect would be limited. This is because plastic film mulching, which had been adopted during the entire winter wheat seasons, can effectively retain soil moisture during the crop growth period [9,10,34]. This could also explain why wheat yield and soil moisture were not substantially affected by GM in the first two experimental years (Figure 1, Figure 2 and Figure 3).

Despite the limited changes observed in the first two years, fertilization improved soil fertility and wheat yields during the third and fourth experimental years (Figure 1). Increased soil nutrient availability facilitates the accumulation of nutrients and photosynthetic assimilates into the fruiting organs or seeds of crops after flowering [23,26]. Wheat straw return further promotes nutrient recycling and increases soil organic carbon content, leading to further improvements in wheat yield [19,35]. Moreover, planting legume GM in the fallow period increased the subsequent winter wheat yield during the third and fourth year, especially the fourth year (Figure 1). This result was consistent with studies showing that GM can enhance crop yield stability and the sustainability of crop production under different circumstances by improving the soil’s fertility, aeration, structure, and health [21,36,37,38]. Legumes can also fix atmospheric N and release it into the soil as mineral N via rhizodeposition and residue decomposition [37,39]. However, the increase in wheat yields due to GM was notably lower than that due to chemical fertilization (Figure 1). This must be due to the fact that the annual N input derived from planting GM (81.9 kg ha^−1^ yr^−1^ on average) was less than that from chemical N fertilization (135 kg ha^−1^ yr^−1^), implying that planting legume GM could not completely replace chemical fertilizer application (Table 4). In summary, wheat yield was increased by chemical fertilization alone and in combination with straw return in the third and fourth experimental years, and planting legume GM during the summer fallow period could also benefit the yield of subsequent winter wheat.

While GM cultivation has commonly demonstrated yield-enhancing effects on subsequent crops in paddy soils and water-sufficient agroecosystems [22,23,38,40], its performance remains contentious in other conditions, especially in northern China’s dryland farming systems, showing either yield reduction or neutral effects on subsequent cereal crops [14,30,31]. A comprehensive meta-analysis identified the key limited factors, including northern China’s semi-arid climate, crop species and planting systems, and especially the region’s limited precipitation, which leads to competition between GM and crops for available water resources [21]. These findings underscore soil water availability as the primary determinant of crop response to both fertilization strategies and agronomic interventions in water-limited environments. However, our experimental design in the Loess Plateau eco-region specifically addressed this hydrological challenge through continuous plastic film mulching during the wheat growing seasons. Therefore, while the third and fourth years had normal precipitation and did not experience extreme temperature, winter wheat growth was not restricted and the positive effects of planting GM and fertilization emerged (Figure 1 and Figure 7). It could also explain that winter wheat yield intensely depended on water consumption since the third year, regardless of GM application or fertilization pattern (Figure 5). This result highlights the importance of water conservation for dryland agriculture, especially where fertilization is being optimized.

Wheat yield is determined by yield components, particularly the number of spikes but also the number of kernels per spike and the 1000-grain weight [2,41]. In the present study, the number of kernels per spike ranged from 36 to 44, with 41–47 g per 1000 grains, during the first three experimental years (Table 1). There were no significant differences among the treatments within each year but there were differences between years. This finding was consistent with other studies in the same region [2,41]. Particularly, chemical fertilization and its combination with wheat straw return substantially increased the number of spikes during the third year of wheat cultivation, ranging from 387 × 10^4^ to 481 × 10^4^ ha^−1^ (Table 1). A higher population density of winter wheat corresponded to greater aboveground biomass during the third experimental year. A relatively higher 1000-grain weight and lower population density of winter wheat were observed in our research than in other studies [2,41]. This could be due to the ridge mulching and furrow planting used in our study, which lowered the real area and density of the wheat. Moreover, the application of legume GM can improve chlorophyll content, leaf area index, net photosynthetic rate, stomatal conductance, and transpiration of crop leaves [23,42]. This could explain the increase in the wheat population and 1000-grain weight in the GM treatments in the third experimental year (Table 1). Therefore, we conclude that planting legume GM in the summer fallow period increases the grain weight and population of winter wheat, especially in the long term.

### 3.2. Dynamics of Soil Moisture and WUE

Soil water use is a key factor controlling crop yields in the Loess Plateau [2,6]. Plastic film mulching is an efficient technique to reduce evaporation, drainage, and runoff, thus increasing the water use efficiency of crops. It has been universally practiced in both southern paddy ecosystems and rainfed ecosystems of northern China [6,34,43]. As the drainage and runoff are both assumed to be negligible in the Loess Plateau in northwest China, plastic film mulching primarily reduces non-productive crop evaporation in this area. In our research, soil moisture was primarily affected by rainfall but also corresponded to the differences in water consumption during each wheat growing stage. Planting legume GM did not largely affect the soil moisture in the first three experimental years except after the booting stage of the third year, when GM decreased soil moisture, indicating increased water consumption in this period (Figure 2, Figure 3 and Figure 4). The current understanding suggests that many crops often exhibit little water demand in their early growth stages, with final yield predominantly determined by precipitation during its critical reproductive stages [21]. Therefore, we speculate that planting GM in the fallow period has a negligible effect on the water consumption of the subsequent winter wheat when grown using plastic film mulching.

Optimizing nutrient management can increase winter wheat requirements for both water and nutrients, as dry matter accumulation in the grain is dependent on the transfer of nutrients between organs, especially in the later growing period [41]. In this study, we estimated the apparent water consumption by summing the soil water input (precipitation) and soil water storage reduction throughout the wheat season. The clear differences observed reflected the water demands of winter wheat, with suitable rainfall resulting in reasonable wheat yields. Chemical fertilization, both alone and in combination with straw return, increased the water consumption of winter wheat in the third experimental year, and these effects were more obvious than those when planting legume GM (Figure 4). Moreover, the water consumption of winter wheat corresponded closely with yield (Figure 1 and Figure 4). The higher crop yields and water consumption also contributed to the higher WUE in fertilized treatments in the third experimental year [34,43]. In comparison, planting legume GM had a less obvious effect on the WUE of winter wheat, mainly due to its limited effect on wheat yield in the short term.

### 3.3. N Nutrition

While soil water is primarily influenced by precipitation, soil nutrient content is directly affected by the agronomic practices used in the field. In the present research, multiple agronomic practices influenced soil nutrient content, including chemical fertilizer application, straw return, and planting legume GM in the fallow period. Fertilization greatly affected the N concentration in both the grain and straw of winter wheat across the four experimental years. Grain, as a key storage organ, was more sensitive to the N input rate than straw (Table 2). The amount of straw returned was 9 Mg ha^−1^ with 68.76 kg N ha^−1^; therefore, the chemical fertilization plus straw return treatment generally increased the wheat N concentration compared with chemical fertilization alone (Table 2 and Table 3). The chemical N application successfully increased the N concentration in wheat plants; however, mineral N is often present in excess in soil during the tillering stage but is depleted during the later stages, especially when all chemical N fertilizer is applied as a basal fertilizer for winter wheat [6,44]. For this reason, chemical fertilizer is often combined with organic amendment additions. Organic amendments can provide a variety of organic and inorganic nutrients, can help immobilize the available N in the soil in the early growing stages, and can release N in the later growing stages [15,23,45]. In our study, both the wheat yield and N concentration in the wheat grain were higher under chemical fertilization plus wheat straw return than under chemical fertilization alone during the third and fourth experimental years (Figure 1 and Table 2). These results reflect the changes in soil nutrient availability.

Similar to wheat straw return, the legume GM was planted and incorporated into soil during the fallow period as an organic amendment. Legume GM exploits the natural resources (light, water, and heat) during the fallow period, can input N into the agroecosystem through biological N fixation, and can return multiple organic and inorganic nutrients to the soil [22,23,29]. In our study, legume GM generally provided a higher amount of mineral N to the soil compared with wheat straw return, but a lower amount compared with chemical N fertilizer. Therefore, GM increased the N concentration in winter wheat, but not to the same extent as chemical fertilization alone, during the third and fourth experimental years (Figure 1, Table 2 and Table 3).

In our study, there were large differences in the amount of annual N input among treatments (Table 3). Moreover, both the NupE and NUE varied among treatments, particularly during the third and fourth experimental years, with NupE and NUE generally decreasing with increasing cumulative N input (Table 3 and Table 4). While a low NupE and NUE commonly indicate a low N utilization effectiveness, they can also indicate greater soil N productivity and lower agronomic efficiency of N, respectively [7,46]. For example, a NupE higher than 1 kg kg^−1^ indicates a negative budget of N during a certain wheat season, and a lower NUE indicates a lower economic return from the N input. In our study, neither chemical fertilization alone nor the application of legume GM without any chemical N input could maintain the positive budget of soil N during some of the experimental years. The decrease in NUE caused by increasing N input could be attributed to the medium fertility of the soil; N was not the limiting factor in this agroecosystem. In summary, application of legume GM alone cannot maintain soil N in the long term, but could be optimized by combining it with other N management strategies, including chemical N application (Table 4). In other words, planting legume GM in the fallow period is suitable for dryland agroecosystems in northwest China but only when combined with other fertilization management strategies. This study highlights the positive contribution of legume GM to the yield and water and nutrient utilization effectiveness of subsequent winter wheat in a rainfed ecosystem. However, further investigation under different circumstances is required.

## 4. Materials and Methods

### 4.1. Experimental Site

The experiment was conducted at the Changwu Agricultural and Ecological Experimental Station (35°12′ N, 107°44′ E, altitude of 1220 m a.s.l.), run by the Chinese Academy of Sciences. This site is located in a typical semi-arid area of the southern Loess Plateau, Shaanxi Province, a major winter wheat production area of northwest China. The area has a continental monsoon climate with hot and dry summers and severe cold winters. The mean temperature and sunshine duration are 9.1 °C and 2230 h (1957–2009), respectively, with 171 frost-free days each year. The average annual precipitation is 534 mm (1994–2014), and 50–60% of the mean rainfall occurs from June to September. The agricultural production in this region is completely dependent on natural precipitation.

The soil is a dark loess that is classified as a Cumulic Haplustoll according to USDA soil taxonomy and is both aridic and loamy [15,24]. The soil used in our experiment possesses moderate fertility and high permeability. Initially, the soil in the plow layer (0–20 cm) exhibited the following properties: a clay content of 24%, a silt content of 70%, and a sand content of 6%; a field water-holding capacity of 22.4%; a pH of 8.11; an organic C content of 8.32 g kg^−1^; a total N content of 0.95 g kg^−1^; a total P content of 0.66 g kg^−1^; a mineral N (NO_3_^−^-N + NH_4_^+^-N) content of 13.7 mg kg^−1^; an available P content of 24.6 mg kg^−1^; and an available K content of 150 mg kg^−1^.

The monthly total precipitation and the mean air temperature during the experimental years from July 2016 to June 2020 are shown in Figure 7a, in which the original data was monitored and recorded by the meteorological station of the experimental station and downloaded from the official website platform (http://cwa.cern.ac.cn/ accessed on 29 June 2021). A frost occurred in early April 2018 and lasted for several days just before the winter wheat booting stage (Figure 7b), which was detrimental to the following wheat yield. The annual precipitation was 602, 505, 654, and 606 mm in each of the four years, respectively (Figure 6). As the seedling, wintering, jointing, booting, filling, and maturity stages of winter wheat emerged around 20 October, 1 December, 10 March, 15 April, 15 May, and 15 June, respectively, the distribution of rainfall in the different winter wheat growing stages is shown in Figure 6. The precipitation during the whole winter wheat growing season (from seedling emergence to maturity) accounted for 46%, 40%, 41%, and 38% of the total annual precipitation, respectively, in the 4 experimental years. According to the previously reported drought index judgment [11], the year 2017–2018 was categorized as a dry year and the years 2016–2017 and 2018–2019 were normal years. Because of the relatively higher rainfall during the summer fallow in 2019, which remained in the soil for subsequent winter wheat growth, the year 2019–2020 could also be categorized as a normal year for winter wheat.

### 4.2. Experimental Design

A four-year field experiment was conducted from July 2016 to June 2020. A split-plot design was used to arrange six treatments. The main treatments consisted of two GM planting regimes (G): (1) wheat planting followed by GM planting and incorporation in the summer fallow (GM) and (2) conventional wheat monoculture followed by bare land summer fallow (BL). The sub-plots consisted of three fertilization (F) treatments: (1) control treatment without any chemical fertilizer (Ct); (2) application of chemical N, P, and K as basal fertilizer (B); and (3) application of basal fertilizer plus wheat straw return before wheat seeding (BS). This resulted in six treatments in a complete block design (2 G treatments × 3 F treatments). In the field, there were three replicate plots (3 × 10 m^2^ in size) for each treatment.

Legume GM (Huai bean, *Glycine ussuriensis* Regel et Maack) was seeded at a rate of 90 kg ha^−1^ in each GM plot annually in mid-July. The GM was chopped and incorporated into the soil in late September before winter wheat seeding. For B treatments, 135 kg N ha^−1^ (urea, 46% N), 120 kg P_2_O_5_ ha^−1^ (triple superphosphate [TSP], 13% P_2_O_5_), and 60 kg K_2_O ha^−1^ (potassium sulfate, 54%) were applied. The straw returned to the field was applied at 9000 kg ha^−1^ (local wheat straw, organic carbon content of 42%) in early October annually before wheat seeding. The rate was slightly higher than the local application standard in order to rapidly improve soil fertility. Winter wheat (*T. aestivum* L.) cultivar ‘Changhan 58’ was seeded at 150 kg ha^−1^ after the fertilizers and wheat straw were uniformly incorporated into the soil.

### 4.3. Field Management

In mid-July every experimental year, the whole experimental field was plowed (10–15 cm in depth) with a rotavator to prepare the seedbed for GM, though GM was only planted in GM treatment plots. GM was seeded via broadcast sowing in mid- to late July, depending on the weather conditions. In late September, about 8–9 weeks after GM sowing, fresh GM at the pod-bearing stage was terminated by chopping the plants into small pieces with mowing machinery and then spread evenly on the soil surface of each GM plot.

At the beginning of October just before winter wheat seeding, wheat straw from the previous season was chopped into small pieces (<5 cm) and then spread evenly throughout the S treatment plots. The straw applied in the first year was purchased from a nearby wheat field, and the straw used in the following years was derived from the experimental plots or purchased. All chemical fertilizers were evenly applied in corresponding plots at one time. Then, the whole field was plowed again using a rotavator, which simultaneously incorporated the chopped GM and wheat straw, as well as chemical fertilizers, into the soil to a depth of 10–15 cm.

**Figure 8:**
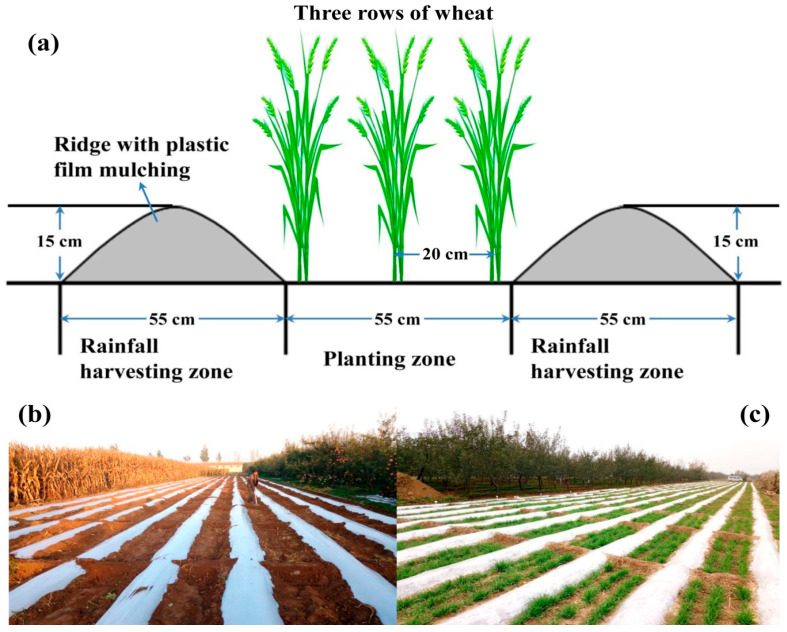
Schematic of the ridge–furrow with the plastic film mulching system (**a**), and with the field performance at the seedbed preparation stage (**b**) and seedling stage (**c**) of winter wheat (cultivar: Changhan 58).

Aiming at water retention in arid soil, the strategy of ridge-mulch with furrow planting was adopted in all treatments during the wheat growing season (Figure 8). After fertilization and plowing, ridges and furrows with a width of 55 cm were hand-formed in each plot. The ridges were all mulched with polyethylene plastic film, and winter wheat was seeded in the furrows and harvested in late June of the following year. After harvesting, almost all the straw was removed and reserved for application in October before wheat seeding. During the four experimental years, winter wheat was sown on 5 October 2016, 1 October 2017, 3 October 2018, and 1 October 2019, and then harvested on 30 June 2017, 30 June 2018, 26 June 2019, and 30 June 2020.

### 4.4. Sampling and Measurements

At the pod-bearing stage of the legume GM in late September, the fresh aboveground biomass of GM was manually collected from two 2 m^2^ areas in the middle of each main plot. After air-drying, the GM samples were oven-dried at 60 °C to determine the biomass content. The aboveground biomass of the legume GM was 4.02, 3.83, 4.45, and 4.58 Mg ha^−1^ in the 2016, 2017, 2018, and 2019 summer fallows, respectively.

Soil samples from 0 to 200 cm depths were collected across the first three growing seasons (2016–2017, 2017–2018, and 2018–2019) of winter wheat at the seedling, wintering, jointing, booting, filling, and maturity stages. The corresponding sampling dates were 15 November, 13 December, 21 March, 30 April, 31 May, and 30 June, respectively, during 2016–2017; 1 November, 16 December, 16 March, 5 May, 5 June, and 30 June during 2017–2018; and 1 November, 15 December, 17 March, 1 May, 3 June, and 26 June during 2018–2019. A 5 cm diameter soil auger was used to collect the soil samples at 20 cm intervals to determine the soil water content. Soil water content in each fresh sample was measured using the drying and weighing method. The soil density (SD) (g cm^−3^) of each soil layer was measured using the core method for each soil layer before wheat sowing in 2018 according to Burgess’s report [47].

The soil water storage at a 0–200 cm depth was calculated using the following equation:

(1) $$SWS=\sum_{i=1}^{n}{SM}_{i}\times {BD}_{i}\times h_{i}\times 0.1$$

where SWS represents the soil water storage at a 0–200 cm depth (mm) at each sampling time; SM*_i_* represents the moisture content (%) in layer *i*; BD*_i_* represents the soil density (g cm^−3^) of layer *i*; h*_i_* represents the depth (cm) of layer *i*; and *i* (1–10) represents the sampling amount per soil profile.

The total water consumption (WC) of winter wheat was calculated as follows:

(2) WC=I+P+ΔSWS+C−R−D

(3) $$\Delta SWS={SWS}_{Before}-{SWS}_{After}$$

where WC represents the cumulative water consumption (mm) during each whole growth season; *I* represents the water amount through irrigation (mm); *P* represents the precipitation (mm) during the whole wheat season; Δ*SWS* represents the soil water storage decrease during the whole season; *C* is the upward flow into the root zone, which can be negligible because the groundwater table remained at a depth of approximately 50–80 m below the surface in this experimental site; and *R* and *D* represent the rainfall losses (mm) through runoff and deep leaching, respectively. Due to the experimental site being located in a typical arid and rainfed farming area, the irrigation (*I*) was zero and the runoff (*R*) and deep leaching (*D*) of the rainfall were negligible. *SWS*_Before_ and *SWS*_After_ represent the soil water storage at a depth of 0–200 cm before and after the entire wheat growth season, respectively.

At harvest time of each year, winter wheat was harvested from two separate 3.3 m^2^ areas (a 3 × 1.1 m^2^ area contained one ridge and one furrow with three wheat rows) in the middle of each plot for yield and yield component determination. The number of spikes per hectare was determined by counting the stems, and 20 spikes per plot were selected for counting the number of kernels per spike. The aboveground biomass, grain yield, and 1000-grain weight were weighed after air-drying. The harvest index (%) was calculated as the total grain weight divided by aboveground biomass. The grain yield and biomass were measured in all four experimental years, but the yield components were only measured during the first three years (2016–2019).

The water use efficiency was calculated using the following equation:

(4) $$WUE=\frac{Y}{WC}$$

where WUE represents the water use efficiency (kg ha^−1^ mm^−1^) of winter wheat during each growth season; Y represents the grain yield (kg ha^−1^) of wheat; and WC represents the total water consumption (mm) during each growth season.

Subsamples of wheat grain and straw from each treatment were collected randomly from the plant samples for laboratory analysis. The grain and straw were separated, oven-dried, and ground into powder to determine the concentration of N. After digesting the dried crop powder with the H_2_SO_4_-H_2_O_2_ method, the N concentration was determined using the Kjeldahl digestion method [48].

N use efficiency included the N uptake efficiency (NupE) and N use efficiency (NUE), which were calculated as described by Guo et al. [46]. The formulas are as follows:

(5) NupE (kg kg−1) = Total N uptakeCumulative N input

(6) NUE (kg kg−1)=Grain yieldTotal N uptake

where Total N uptake represents the total N amount present in the aboveground biomass (both grain and straw) of winter wheat and the cumulative N input represents the total N input derived from all agronomic and natural strategies. In addition to the effect of chemical N fertilizer input on the current wheat, we also estimated the N inputs from other sources, including the rated straw return, the remaining wheat stubble from the preceding year, the legume GM seed as well as its growth and incorporation, the wheat seed, and the atmospheric deposition. In this study, it was determined that returned wheat straw contained 0.76% N on average. The average proportion of wheat stubble that was left and remained in the field during harvesting to total straw was estimated to be 15%, and the N concentrations from various treatments were determined separately. The biomass-C derived from the aboveground and belowground (root + rhizodeposition) portions was estimated to be 75.9% and 24.1% of the net primary productivity of legume GM, which was calculated according to Bolinder’s study [49]. The N concentrations in aboveground and belowground biomass were determined as 1.4% and 1.7%, respectively, on average. The wheat seed contained 2.2% N. The average N input derived from atmospheric deposition was estimated to be 21.76 kg ha^−1^ annually, according to Liang’s report [50].

### 4.5. Statistical Analysis

Statistical analyses were performed using the Windows-based Statistical Package for the Social Sciences program (SPSS v19.0). Both the normality of distribution and constant error variance assumptions were tested for data analysis. Data were subjected to analysis of variance (ANOVA) for each year to evaluate the effects of the planting regime (G), fertilization treatment (F), and their interaction (G × F) on all measured parameters. Treatment means were compared using the least significant difference test (LSD), and *p* < 0.05 was regarded as statistically significant. G and F had little or no significant effect on soil water content, and thus the soil water content was visually compared among different treatments. Pearson correlation analysis was used to determine the relationships between the grain yield of winter wheat and water consumption (WC) in different experimental years. Figures were produced using Origin Pro 2022 (V.2022).

## 5. Conclusions

Our short-term research demonstrated that planting legume GM in the summer fallow period seems not to decrease the soil moisture and water storage during the subsequent winter wheat period when wheat is grown using plastic film mulching even in dry years in the Loess Plateau of China. Conversely, planting legume GM tended to improve the yield of winter wheat and improve its water use efficiency in the long term, especially in years with normal precipitation and temperature. Compared with planting legume GM, chemical fertilizer application alone and coupled with straw return could more substantially improve the yield of winter wheat, primarily by increasing the population density and water use efficiency. When integrated with chemical fertilization and straw return, planting legume GM further improved soil N sustainability for winter wheat production. Our findings provide insights into the use of legume GM in the fallow period to optimize agronomic practices and make full use of soil water and nutrients in a rainfed agroecosystem.

## Figures and Tables

**Table 1:** Aboveground biomass, spikes per hectare, number of kernels per spike, 1000-grain weight, and harvest index of winter wheat as affected by planting regimes (G) and fertilization patterns (F) during the four experimental years.

Experimental Year	Treatment	Aboveground Biomass (Mg ha^−1^)	Spikes ha^−1^ (×10^4^)	Kernels Spike^−1^	1000-Grain Weight (g)	Harvest Index
2016–2017	BL-Ct	14.06 ± 0.14 c	434 ± 9 b	39.4 ± 0.8 a	42.4 ± 0.5 a	0.37 ± 0.01 a
	BL-B	17.24 ± 0.27 a	397 ± 11 c	39.0 ± 0.5 a	43.2 ± 0.7 a	0.28 ± 0.01 c
	BL-BS	16.24 ± 0.10 b	464 ± 6 a	40.7 ± 0.8 a	42.3 ± 0.6 a	0.35 ± 0.01 b
	Mean	15.85 ± 0.48 A	432 ± 11 A	39.7 ± 0.4 A	42.7 ± 0.3 A	0.33 ± 0.01 A
	GM-Ct	15.93 ± 0.45 a	447 ± 10 a	40.2 ± 0.6 a	42.4 ± 0.6 a	0.34 ± 0.01 a
	GM-B	15.26 ± 0.21 a	349 ± 13 c	39.7 ± 0.5 a	43.4 ± 0.7 a	0.30 ± 0.01 b
	GM-BS	14.44 ± 0.17 b	384 ± 9 b	39.9 ± 0.5 a	43.3 ± 0.5 a	0.34 ± 0.01 a
	Mean	15.21 ± 0.26 A	393 ± 15 A	39.9 ± 0.3 A	43.0 ± 0.3 A	0.33 ± 0.01 A
	ANOVA *p* value					
	G	0.088	0.067	0.777	0.284	0.414
	F	<0.001	<0.001	0.343	0.500	<0.001
	G×F	0.000	0.003	0.376	0.793	0.002
						
2017–2018	BL-Ct	9.72 ± 0.21 b	356 ± 6 b	36.1 ± 0.6 a	41.1 ± 0.6 a	0.41 ± 0.01 b
	BL-B	10.01 ± 0.20 b	393 ± 7 a	36.9 ± 0.6 a	41.1 ± 0.7 a	0.45 ± 0.01 a
	BL-BS	10.94 ± 0.13 a	400 ± 5 a	37.1 ± 0.6 a	41.8 ± 0.6 a	0.42 ± 0.01 ab
	Mean	10.22 ± 0.21 A	383 ± 7 A	36.7 ± 0.4 A	41.3 ± 0.4 A	0.43 ± 0.01 A
	GM-Ct	10.25 ± 0.18 a	407 ± 6 a	37.1 ± 0.6 a	42.8 ± 0.6 a	0.46 ± 0.02 a
	GM-B	8.75 ± 0.24 b	277 ± 18 c	36.3 ± 1.1 a	41.3 ± 0.7 a	0.36 ± 0.02 c
	GM-BS	8.78 ± 0.26 b	312 ± 17 b	36.3 ± 1.0 a	41.7 ± 0.6 a	0.41 ± 0.02 b
	Mean	9.26 ± 0.27 B	332 ± 21 B	36.6 ± 0.5 A	41.9 ± 0.4 A	0.41 ± 0.02 A
	ANOVA *p* value					
	G	0.004	0.041	0.876	0.331	0.440
	F	0.008	0.003	0.986	0.475	0.059
	G×F	<0.001	<0.001	0.484	0.347	0.001
						
2018–2019	BL-Ct	11.76 ± 0.38 b	387 ± 8 c	42.7 ± 0.6 b	45.5 ± 0.1 b	0.41 ± 0.01 a
	BL-B	13.47 ± 0.14 a	448 ± 9 b	43.4 ± 0.7 ab	46.5 ± 0.4 a	0.42 ± 0.01 a
	BL-BS	14.40 ± 0.49 a	477 ± 7 a	44.4 ± 0.6 a	46.2 ± 0.5 a	0.42 ± 0.01 a
	Mean	13.21 ± 0.43 A	437 ± 14 A	43.5 ± 0.4 A	46.0 ± 0.2 A	0.42 ± 0.01 A
	GM-Ct	13.19 ± 0.35 c	409 ± 7 c	43.3 ± 0.6 a	46.2 ± 0.4 b	0.39 ± 0.01 a
	GM-B	14.44 ± 0.26 b	452 ± 5 b	44.3 ± 0.8 a	47.3 ± 0.4 a	0.40 ± 0.01 a
	GM-BS	15.89 ± 0.32 a	481 ± 9 a	44.4 ± 0.8 a	47.5 ± 0.5 a	0.39 ± 0.01 a
	Mean	14.51 ± 0.42 A	447 ± 11 A	44.0 ± 0.4 A	47.0 ± 0.3 A	0.39 ± 0.01 A
	ANOVA *p* value					
	G	0.075	0.355	0.294	0.260	0.056
	F	<0.001	<0.001	0.074	0.019	0.253
	G×F	0.697	0.393	0.691	0.654	0.437

Ct, B, and BS represent the control (no fertilization), basal fertilization with NPK fertilizers, and basal fertilization plus wheat straw return, respectively. GM represents planting GM and incorporation during summer fallow before the wheat season, compared with conventional wheat cultivation after a bare land fallow (BL). Two-way (G and F) ANOVA and an LSD test were used for each item to determine the significances among treatments. Data are shown as mean ± SE (n = 3). Different upper-case letters indicate a significant difference (*p* < 0.05) between the two main treatments affected by G. Different lower-case letters indicate significant differences (*p* < 0.05) between the three fertilization patterns.

**Table 2:** The N concentrations (g kg^−1^) in the grain and straw of winter wheat as affected by planting regimes (G) and fertilization patterns (F) during the four experimental years.

Treatment	2016–2017			2017–2018			2018–2019			2019–2020	
	Grain	Straw		Grain	Straw		Grain	Straw		Grain	Straw
BL-Ct	20.53 ± 0.36 a	7.20 ± 0.23 b		20.10 ± 0.37 b	6.05 ± 0.14 b		21.70 ± 0.19 b	5.77 ± 0.05 b		21.10 ± 0.23 b	6.00 ± 0.09 c
BL-B	20.70 ± 0.08 a	9.09 ± 0.19 a		20.52 ± 0.24 b	7.42 ± 0.17 a		22.29 ± 0.25 ab	6.50 ± 0.17 a		21.60 ± 0.18 b	7.03 ± 0.10 a
BL-BS	21.09 ± 0.14 a	8.94 ± 0.11 a		21.43 ± 0.33 a	7.21 ± 0.08 a		23.26 ± 0.48 a	6.05 ± 0.08 b		22.49 ± 0.37 a	6.69 ± 0.05 b
Mean	20.77 ± 0.14 A	8.41 ± 0.32 B		20.68 ± 0.25 B	6.89 ± 0.22 A		22.41 ± 0.28 B	6.11 ± 0.12 B		21.73 ± 0.24 B	6.57 ± 0.16 B
GM-Ct	20.78 ± 0.19 b	9.08 ± 0.26 b		20.34 ± 0.16 c	6.23 ± 0.11 c		22.63 ± 0.29 c	5.52 ± 0.19 c		21.75 ± 0.12 c	6.09 ± 0.08 c
GM-B	20.82 ± 0.39 b	9.35 ± 0.13 ab		21.76 ± 0.25 b	7.18 ± 0.09 b		23.83 ± 0.68 b	6.77 ± 0.11 b		22.91 ± 0.45 b	7.15 ± 0.07 b
GM-BS	22.09 ± 0.11 a	9.82 ± 0.26 a		23.44 ± 0.03 a	7.74 ± 0.14 a		25.11 ± 0.28 a	7.95 ± 0.06 a		24.30 ± 0.18 a	8.07 ± 0.08 a
Mean	21.23 ± 0.25 A	9.42 ± 0.15 A		21.84 ± 0.46 A	7.05 ± 0.23 A		23.86 ± 0.43 A	6.74 ± 0.36 A		22.99 ± 0.40 A	7.10 ± 0.29 A
ANOVA *p* value											
G	0.222	0.039		0.018	0.248		0.035	0.023		0.030	0.027
F	0.005	0.001		<0.001	<0.001		0.007	<0.001		0.001	<0.001
G×F	0.162	0.012		0.049	0.037		0.600	<0.001		0.227	<0.001

Note: Ct, B, and BS represent the control (no fertilization), basal fertilization with NPK fertilizers, and basal fertilization plus wheat straw return, respectively. GM represents planting GM and incorporation during summer fallow before the wheat season, compared with conventional wheat cultivation after a bare land fallow (BL). Two-way (G and F) ANOVA and an LSD test were used for each item to determine the significances among treatments. Data are shown as mean ± SE (n = 3). Different upper-case letters indicate a significant difference (*p* < 0.05) between the two main treatments affected by G. Different lower-case letters indicate significant differences (*p* < 0.05) between the three fertilization patterns.

**Table 3:** Estimates of cumulative N inputs (kg ha^−1^) from multiple resources among different treatments during the four experimental years.

Experimental Year	Treatment	GM-Derived			Wheat Straw-Derived			N Fertilizer		Wheat Seed		Atmospheric Deposition		Total
		Shoot	Root + Rhizodeposition		Preceding Stubble	Rated Straw Return								
2016–2017	BL-Ct	0	0		0	0		0		3.30		21.76		25.1
	BL-B	0	0		0	0		135		3.30		21.76		160
	BL-BS	0	0		0	68.76		135		3.30		21.76		229
	GM-Ct	56.28	21.70		0	0		0		3.30		21.76		103
	GM-B	56.28	21.70		0	0		135		3.30		21.76		238
	GM-BS	56.28	21.70		0	68.76		135		3.30		21.76		307
														
2017–2018	BL-Ct	0	0		9.59	0		0		3.30		21.76		34.7
	BL-B	0	0		16.89	0		135		3.30		21.76		177
	BL-BS	0	0		14.26	68.76		135		3.30		21.76		243
	GM-Ct	53.62	20.67		14.34	0		0		3.30		21.76		114
	GM-B	53.62	20.67		15.00	0		135		3.30		21.76		249
	GM-BS	53.62	20.67		14.10	68.76		135		3.30		21.76		317
														
2018–2019	BL-Ct	0	0		5.18	0		0		3.30		21.76		30.2
	BL-B	0	0		6.17	0		135		3.30		21.76		166
	BL-BS	0	0		6.90	68.76		135		3.30		21.76		236
	GM-Ct	62.30	24.02		5.21	0		0		3.30		21.76		117
	GM-B	62.30	24.02		6.00	0		135		3.30		21.76		252
	GM-BS	62.30	24.02		6.04	68.76		135		3.30		21.76		321
														
2019–2020	BL-Ct	0	0		6.01	0		0		3.30		21.76		31.1
	BL-B	0	0		7.65	0		135		3.30		21.76		168
	BL-BS	0	0		7.86	68.76		135		3.30		21.76		237
	GM-Ct	64.12	24.72		6.69	0		0		3.30		21.76		121
	GM-B	64.12	24.72		8.83	0		135		3.30		21.76		258
	GM-BS	64.12	24.72		11.63	68.76		135		3.30		21.76		329

Note: Ct, B, and BS represent the control (no fertilization), basal fertilization with NPK fertilizers, and basal fertilization plus wheat straw return, respectively. GM represents planting GM and incorporation during summer fallow before the wheat season, compared with conventional wheat cultivation after a bare land fallow (BL).

**Table 4:** Nitrogen uptake efficiency (NupE, kg kg^−1^) and nitrogen use efficiency (NUE, kg kg^−1^) of winter wheat as affected by planting regimes (G) and fertilization patterns (F) during the four experimental years.

Treatment	2016–2017			2017–2018			2018–2019			2019–2020	
	NupE	NUE		NupE	NUE		NupE	NUE		NupE	NUE
BL-Ct	6.78 ± 0.07 a	30.4 ± 0.60 a		3.33 ± 0.13 a	34.9 ± 0.83 a		4.78 ± 0.15 a	33.3 ± 0.26 a		4.94 ± 0.20 a	33.5 ± 0.14 a
BL-B	1.33 ± 0.02 b	22.9 ± 0.83 c		0.75 ± 0.02 b	33.6 ± 0.54 a		1.06 ± 0.01 b	31.9 ± 0.08 b		1.12 ± 0.02 b	31.3 ± 0.33 b
BL-BS	0.93 ± 0.01 c	26.3 ± 0.40 b		0.59 ± 0.01 c	31.7 ± 0.71 b		0.81 ± 0.03 c	31.3 ± 0.49 b		0.85 ± 0.01 c	31.1 ± 0.76 b
Mean	3.02 ± 0.94 A	26.5 ± 1.14 A		1.56 ± 0.44 A	33.4 ± 0.58 A		2.22 ± 0.64 A	32.2 ± 0.35 A		2.31 ± 0.66 A	31.9 ± 0.46 A
GM-Ct	2.02 ± 0.09 a	26.0 ± 0.27 a		1.14 ± 0.01 a	36.0 ± 0.59 a		1.37 ± 0.03 a	31.9 ± 0.10 a		1.40 ± 0.05 a	32.4 ± 0.05 a
GM-B	0.82 ± 0.01 b	23.4 ± 0.46 b		0.44 ± 0.01 b	29.1 ± 0.87 b		0.78 ± 0.01 b	29.4 ± 0.73 b		0.79 ± 0.02 b	29.8 ± 0.41 b
GM-BS	0.66 ± 0.01 c	24.1 ± 0.28 b		0.39 ± 0.01 b	28.9 ± 0.67 b		0.72 ± 0.01 b	26.5 ± 0.54 c		0.71 ± 0.02 b	27.5 ± 0.28 c
Mean	1.17 ± 0.22 B	24.5 ± 0.43 A		0.66 ± 0.12 B	31.3 ± 1.22 A		0.96 ± 0.10 B	29.3 ± 0.83 B		0.97 ± 0.11 B	29.9 ± 0.71 B
ANOVA *p* value											
G	<0.001	0.083		0.003	0.075		0.002	0.033		0.001	0.033
F	<0.001	<0.001		<0.001	<0.001		<0.001	<0.001		<0.001	<0.001
G×F	<0.001	0.002		<0.001	0.008		0.000	0.003		<0.001	0.026

Note: Ct, B, and BS represent the control (no fertilization), basal fertilization with NPK fertilizers, and basal fertilization plus wheat straw return, respectively. GM represents planting GM and incorporation during summer fallow before the wheat season, compared with conventional wheat cultivation after a bare land fallow (BL). Two-way (G and F) ANOVA and an LSD test were used for each item to determine the significances among treatments. Data are shown as mean ± SE (n = 3). Different upper-case letters indicate a significant difference (*p* < 0.05) between the two main treatments affected by G. Different lower-case letters indicate significant differences (*p* < 0.05) between the three fertilization patterns.

## Data Availability

All relevant data are within the paper.
